# The role of an in-house audit group as an innovative tool to review clinical trials

**DOI:** 10.1186/1745-6215-16-S2-O50

**Published:** 2015-11-16

**Authors:** Shivali Trivedi, Krishna Hathi, Natasha Aslam, Nausheen Saleem, Kylie Gyertson, Jeremy Whelan

**Affiliations:** 1 University College London Hospitals, London, UK

## Background

Auditing is an integral part of quality assurance in trials. The introduction of risk-based monitoring in industry coupled with reduced funding for academic trials indicates a need to enhance the comprehensive oversight of trials. Generating an internal audit system streamlines quality management across the clinical trials unit. We aimed to pilot a robust in-house process that prioritises patient safety and monitors trial delivery.

## Methods

Ten healthcare professionals within our team voluntarily formed an internal audit group and received appropriate training. Sub-teams were assigned individual trials to audit over a three month period. Quality and SOP compliance data was collated in a comprehensive audit spreadsheet and processes covered included examination of Site Files, Case Report Forms, Serious Adverse Events (SAE), and source data verification (SDV). Findings were amalgamated into a Corrective Action Preventative Action (CAPA) Plan and fed back to the wider team. Team actions were subsequently reviewed after six weeks as a follow-up measure.

## Results

Preliminary findings of five internal audits highlighted areas for improvement within data and procedural systems including; informed consent, SAE reporting, and SDV. Additionally, the Audit Group proved a useful measure in developing overall quality standards of the team, highlighted by staff learning, improved knowledge and adherence to regulatory standards.

## Conclusions

In line with upcoming EU legislation and ICH GCP, maintaining rigorous auditing processes and adherence to quality standards is of utmost importance. The ongoing quarterly internal audit cycle will be continuously reviewed, feeding into an evolving quality management system ensuring best practice across the unit.

